# Supplementary material for: A powder-metallurgy-based strategy toward three-dimensional graphene-like network for reinforcing copper matrix composites
Source: Nat Commun. 2020 Jun 2;11:2775. doi: 10.1038/s41467-020-16490-4 (PMC7265306; doi:10.1038/s41467-020-16490-4)
Supplement: Supplementary file 1 — Supplementary Information [file 41467_2020_16490_MOESM1_ESM.pdf]

Supplementary Information

A powder-metallurgy-based strategy toward three-dimensional graphene-like network for reinforcing copper matrix composites

Zhang et al.

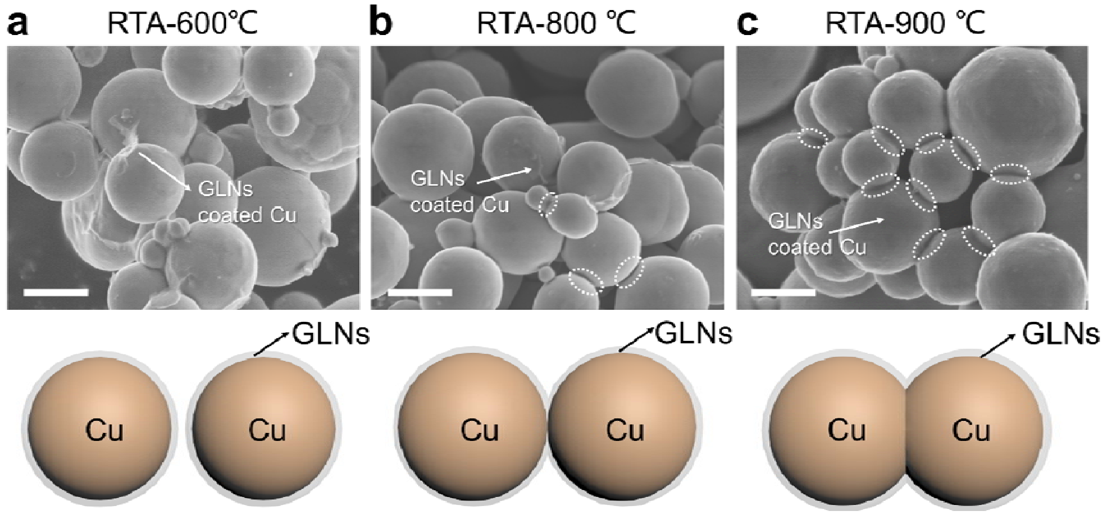

Supplementary Figure 1. SEM images and illustrations of the GLNs/Cu powder. **a** RTA-600°C. **b** RTA-800°C. **c** RTA-900°C. With the increase of the temperature, the Cu powders had an obvious tendency to be sintered together and lost the powder character at a temperature >900°C. Scale bar, 1 μm.

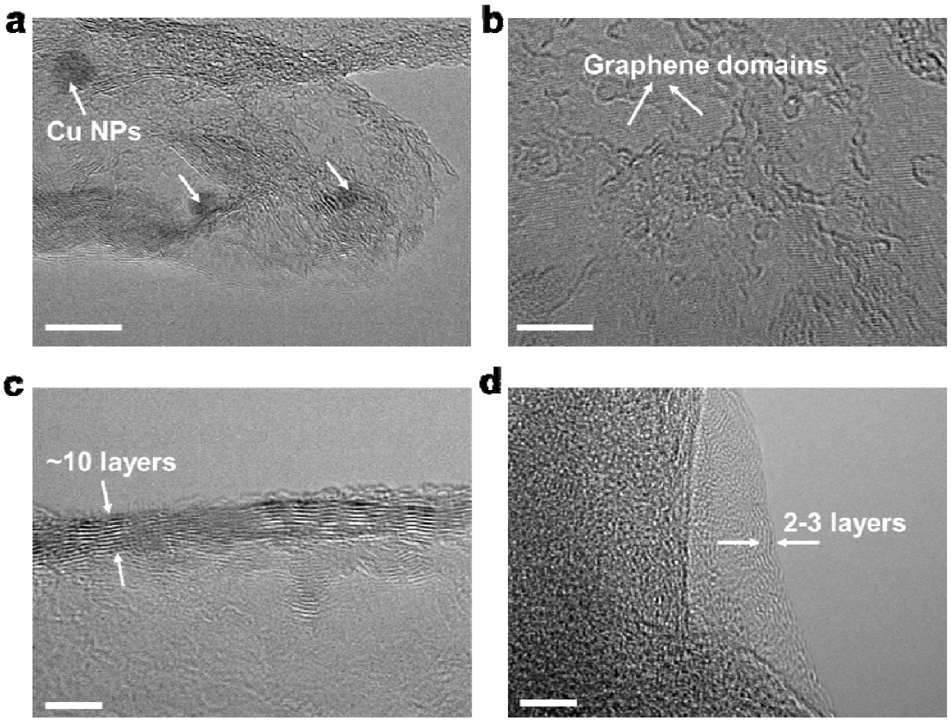

Supplementary Figure 2. Detailed HRTEM characterization of GLNs. a Edge area with thin-thickness feature. Scale bar, 10 nm. **b** Graphene domains (~5 nm) and amorphous carbon morphology. **c**,**d**, Edges of different graphene layers (**c**) ~10 layers and (**d**) 2-3 layers. Scale bar, 5 nm (**b-d**).

Supplementary Note 1. Discussion on the distribution of RGO nanosheets in the Cu matrix

In order to comparatively analyze the distinctive distribution of 3D-GLNN, RGO which is a typical 2D derivative of graphene, was used as the initial material and fabricated into bulk composites with a similar series of processing steps with 3D-GLNN/Cu for comparison. It was found that RGO nanosheets with a typical geometric size of about 2-5 μm dispersed on the partial surface of Cu powders rather than formed an intact coating structure like GLNs/Cu (Supplementary Figure 3a-d). Despite of the fact the two types of fillers shared a similar distribution characteristics at the grain boundary position, it turned out that RGO nanosheets failed in constructing a continuous network and stacked into agglomerates somewhere after hot-pressing (Supplementary Figure 3e and f). Even the severe plastic deformation during hot-rolling was incapable to break up the agglomerates formed by the larger shaped RGO nanosheets but aligned some of smaller ones to the rolling direction (Supplementary Figure 3g and i).

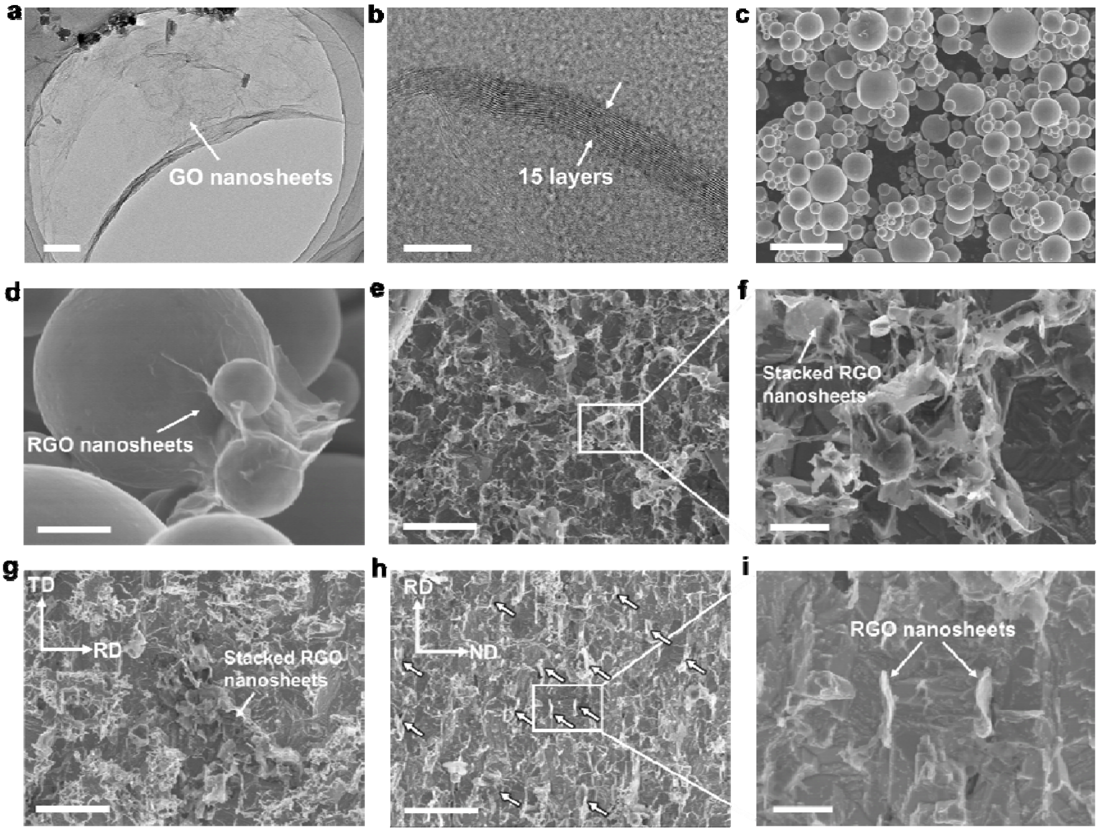

Supplementary Figure 3. Typical morphology characterizations of GO and RGO/Cu. a,b, TEM image showing the general morphology of GO (**a**) and the corresponding edge area (**b**). Scale bar, 200 nm (**a**); 10 nm (**b**)**. c**,**d**, SEM images of RGO/Cu powders showing the general morphology of Cu powders (**c**) and RGO nanosheets decorated on the surface of Cu powders (**d**) after heat-treatment at 800°C. Scale bar, 5 μm (**c**); 500 nm (**d**). **e**,**f**, SEM image of RGO exposed from the etched surface of hot-pressed RGO/Cu (**e**) and the corresponding enlarged view of the marked area (**f**). Scale bar, 5 μm (**e**); 1 μm (**f**). **g** Hot-rolled RGO/Cu in the TD-RD plane, showing the stacked RGO nanosheets in the bulk composites. Scale bar, 5 μm. **h**,**i,** SEM image of hot-rolled RGO/Cu in the RD-ND plane (**h**) and the corresponding enlarged view of the marked area (**i**). Scale bar, 5 μm (**h**); 1 μm (**i**).

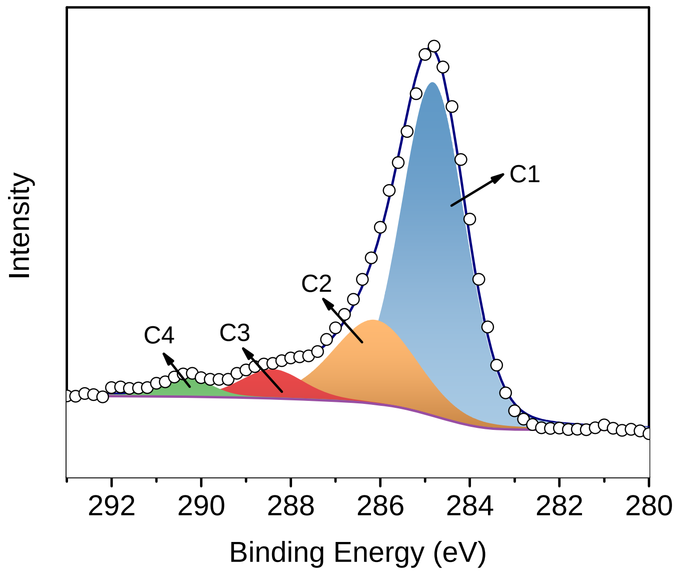

Supplementary Figure 4. Deconvoluted XPS C1s spectrum of 400-RTA composite powders.

Supplementary Table 1. The XPS and Raman spectra analysis data.

Analysis type			XPS			Raman
Samples	p_{sp^2} (%)	p_{sp^3} (%)	$p_{C\text{–}O}$ (%)	$p_{C=O}$ (%)	p_{sp^3} / p_{sp^2}	I_D / I_G
400-RTA	52.5	27.9	15.4	4.1	0.53	0.99
800-RTA	75.3	14.1	7.0	3.6	0.19	0.53
800-800-50	90.1	6.1	2.2	0.9	0.07	0.63
800-800-0	43.8	36.2	13.8	6.2	0.83	0.87
800-400-50	3.2	45.1	46.8	5.0	14.3	0.85
400-800-50	85.7	9.5	2.8	2.0	0.11	0.88

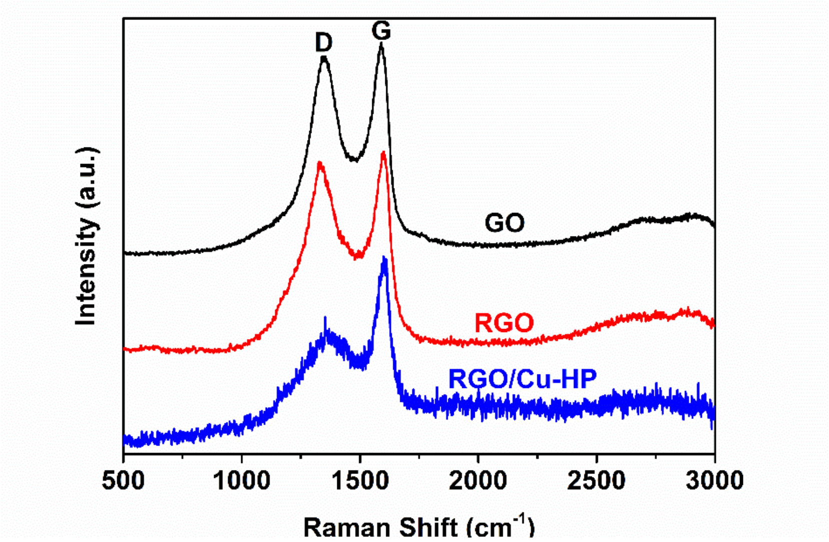

Supplementary Figure 5. Raman spectra of GO, RGO and RGO/Cu-HP.

Supplementary Note 2. Discussion of the shape and size effect of Cu powders on the formation of 3D-GLNN.

Different Cu coppers, namely irregular Cu powders with an average of size of 5 μm (5R-Cu) and spherical Cu powders with an average diameter of 40 μm (40S-Cu), were used as the template and catalyst to synthesize GLNs on their surface under the same preparation conditions. Macroscopically, some of the 5R-Cu powders were partially sintered together (Supplementary Figure 6a, b), while only isolated powder morphology was found for GLNs/40S-Cu powders after annealing at 800°C (Supplementary Figure 6d). And it turned out that for both cases GLNs were grown homogeneously on the Cu powder surface (Supplementary Figure 6b, c, e and f). To further elucidate the influence of different Cu powders on the formation of 3D-GLNN, we prepared two groups of hot-pressed samples with 5R-Cu and 40S-Cu powders, respectively. It could be seen form Supplementary Figure 7a that an incomplete and irregular network structure exposed after surface etching with etching agent. The magnified SEM images in Supplementary Figure 7b, c validated that some of the GLNs were fractured after hot-pressing which could be attributed to the reason of nonuniform thermal stress caused by the irregular shape of Cu powders. While for GLNs/40S-Cu-HP, despite of the successful formation of a continuous network structure in Cu matrix, numbers of voids were spotted in the junction area (as shown in Supplementary Figure 7d-f). It is suggested that the full encapsulation of graphene separated the 40S-Cu powders from forming effective sintering necks during hot-pressing so that the Cu matrix is not continuous in the bulk composites. As

discussed above, the successful construction of 3D-GLNN/Cu requires the Cu powders to be in regular spherical shape and have relatively small size to facilitate partial sintering between Cu powders during GLNs synthesis and thus form the interpenetrating graphene-like network structure afterwards.

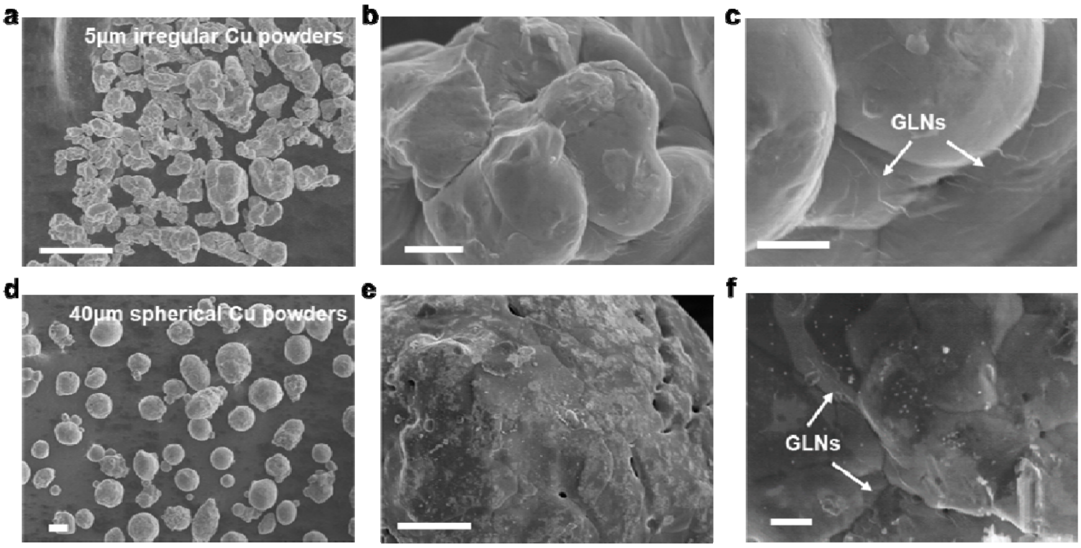

Supplementary Figure 6. SEM images of GLNs/Cu hybrid powders fabricated by different Cu powders. a-c Morphology of 5R-Cu after GLNs growth (**a**), the partial-sintering morphology of 5R-Cu (**b**) and the magnified image showing GLNs grown on the surface of 5R-Cu (**c**). Scale bar, 20 μm (**a**); 1 μm (**b**); 500 nm (**c**). **d-f** Isolated powder morphology of 40S-Cu (**d**) and the relatively smooth surface of 40S-Cu after GLNs growth (**e**), and (**f**) the magnified image showing GLNs grown on the surface of 40S-Cu. Scale bar, 20 μm (**d**); 5 μm (**e**); 500 nm (**f**).

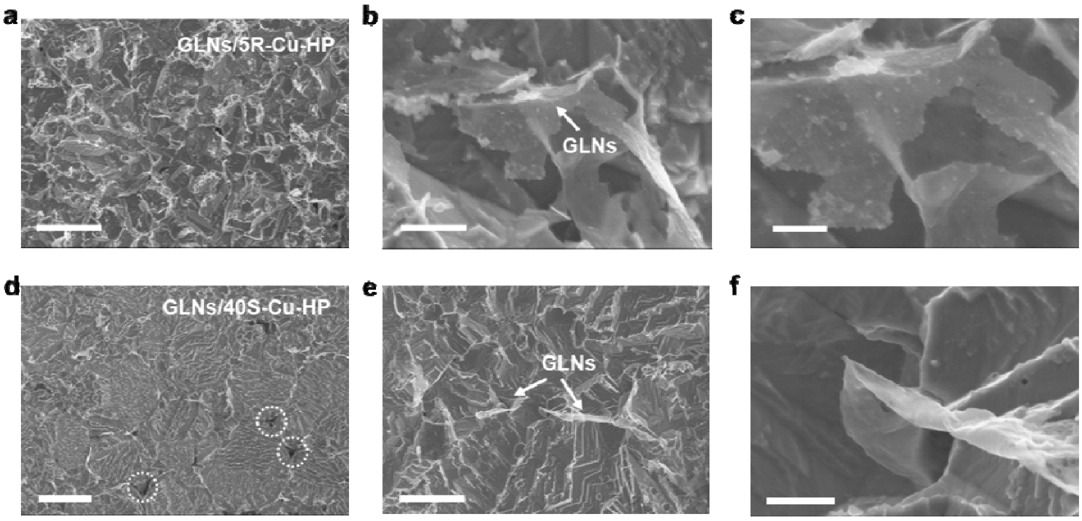

Supplementary Figure 7. SEM images of the 3D-GLNNs exposed from the etched bulk surface. a-c Incomplete and irregular network structure exposed after surface etching of GLNs/5R-Cu-HP (**a**), typical morphology of the isolated GLNs in GLNs/5R-Cu-HP (**b**) and the corresponding enlarged view (**c**). Scale bar, 5 μm (**a**); 500 nm (**b**); 200 nm (**c**). **d-f** Regular network structure exposed after surface etching of GLNs/40S-HP (**d**), typical morphology of GLNs in GLNs/40S-HP (**e**) and the corresponding enlarged view (**f**). Scale bar, 20 μm (**d**); 5 μm (**e**); 500 nm (**f**).

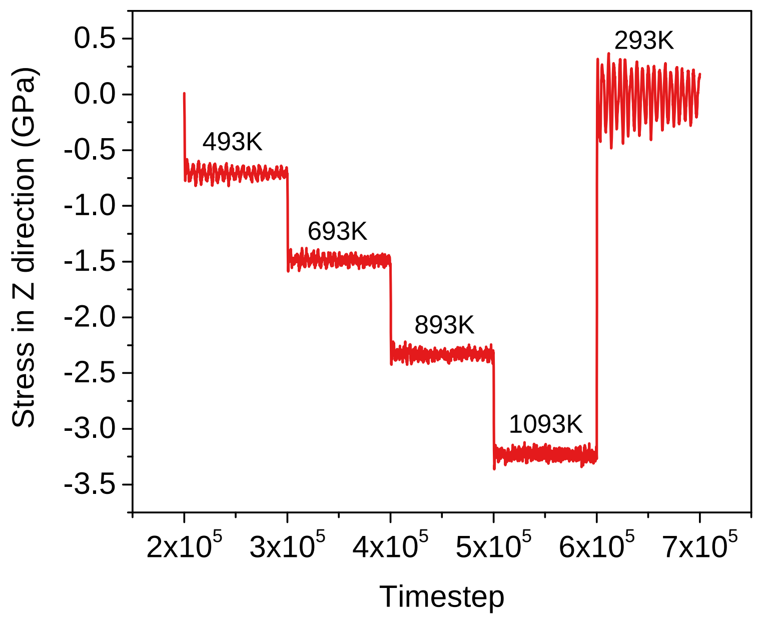

Supplementary Figure 8. The total stress in Z direction of 6LGs/Cu at different temperatures.

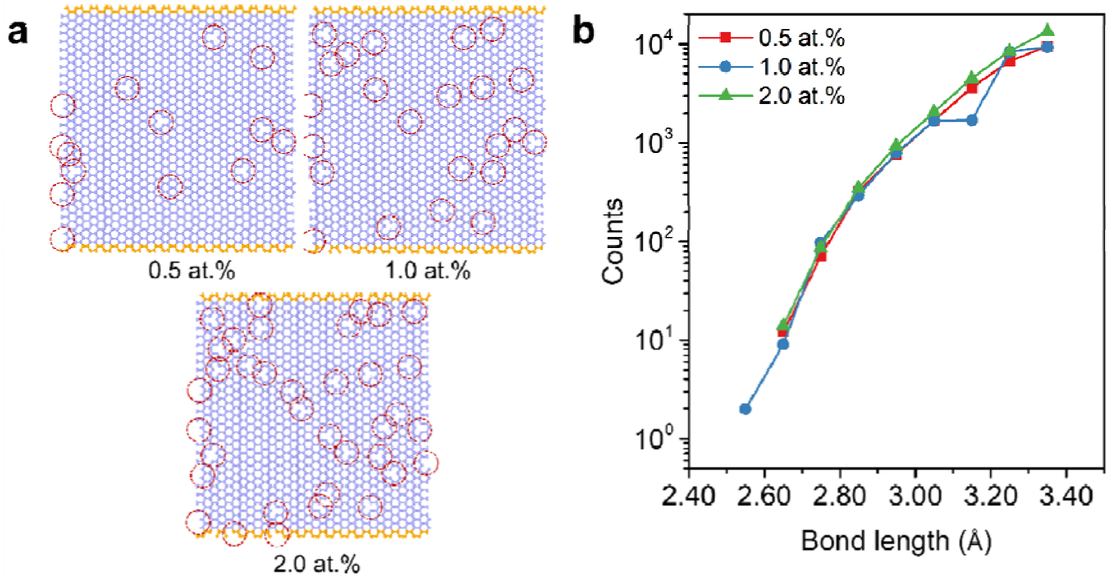

Supplementary Figure 9. MD simulations on defected graphene/Cu models. a Atomic configurations of graphene with different concentrations of single-vacancy defects. **b** The bond length statistics of 6LGs/Cu at 1093K with different defect concentrations.

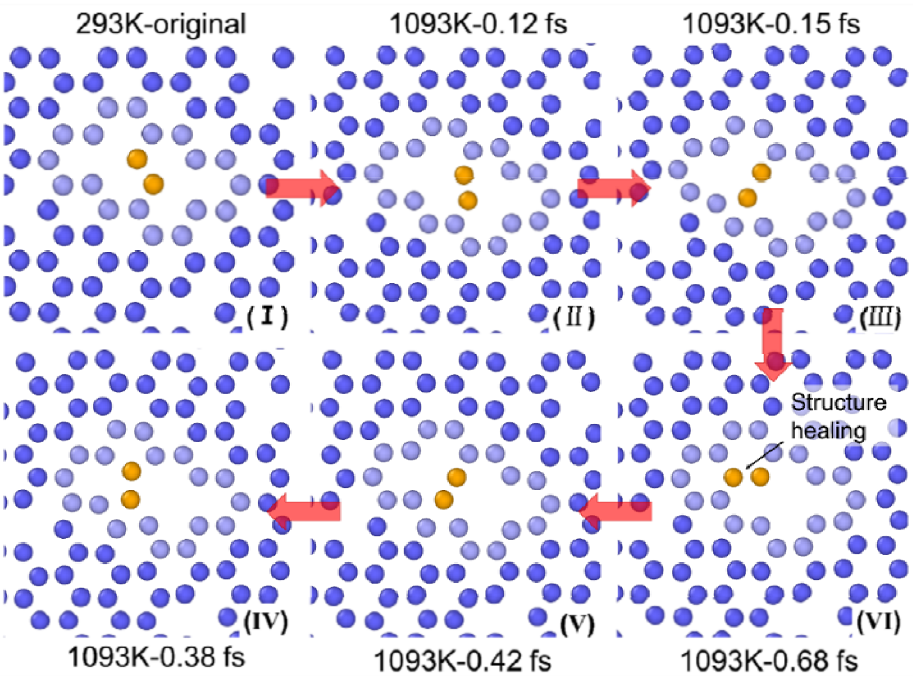

Supplementary Figure 10. A typical structural healing progress in the graphene layer close to the Cu matrix in 6LGs/Cu at 1093K.

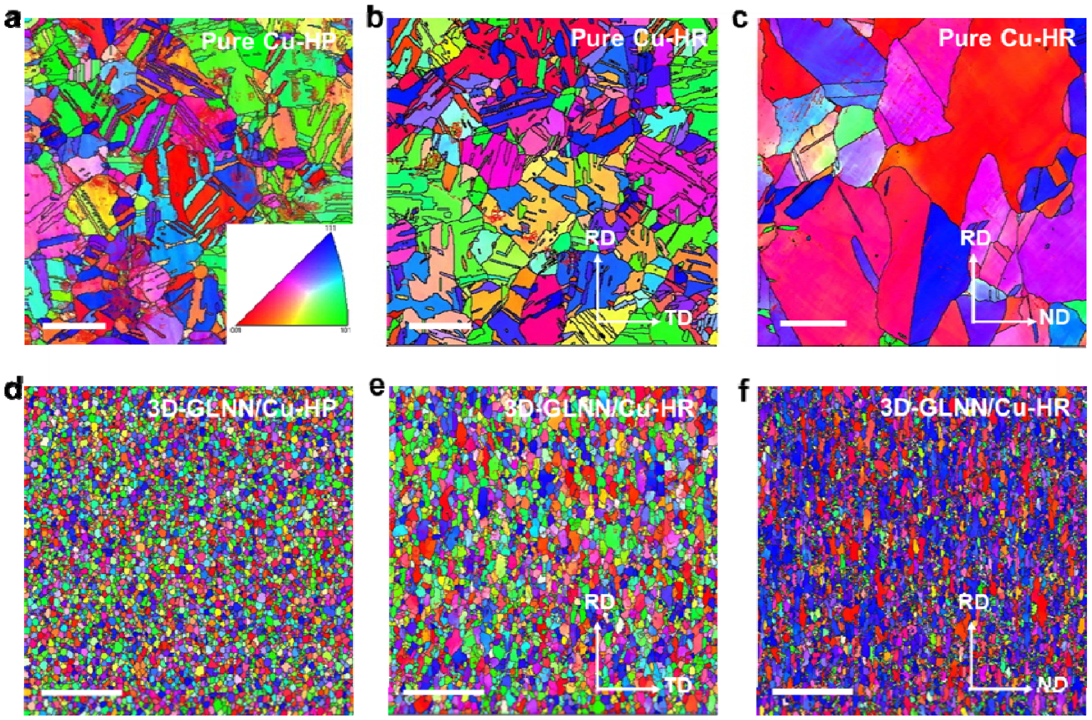

Supplementary Figure 11. EBSD micrographs of pure Cu and 3D-GLNN/Cu. a Pure Cu-HP. Scale bar, 100 μm. **b** Pure Cu-HR from RD-TD plane. Scale bar, 200 μm. **c** Pure Cu-HR from RD-ND plane. Scale bar, 50 μm. **d** 3D-GLNN/Cu-HP. **e** 3D-GLNN/Cu-HR from RD-TD plane. **f** 3D-GLNN/Cu-HR from RD-ND plane. Scale bar, 20 μm (**d**-**f**).

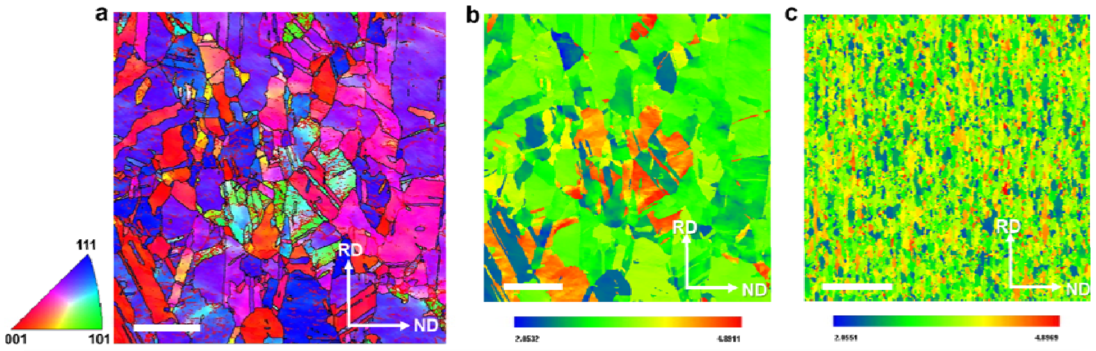

Supplementary Figure 12. EBSD micrographs and the corresponding Taylor factor maps. a RGO/Cu-HR from RD-ND plane. Scale bar, 50 μm. **b,c,** Taylor factor maps of RGO/Cu-HR from RD-ND plane (**b**) and 3D-GLNN/Cu-HR from RD-ND plane (**c**). Scale bar, 50 μm (**b**); 20 μm (**c**).

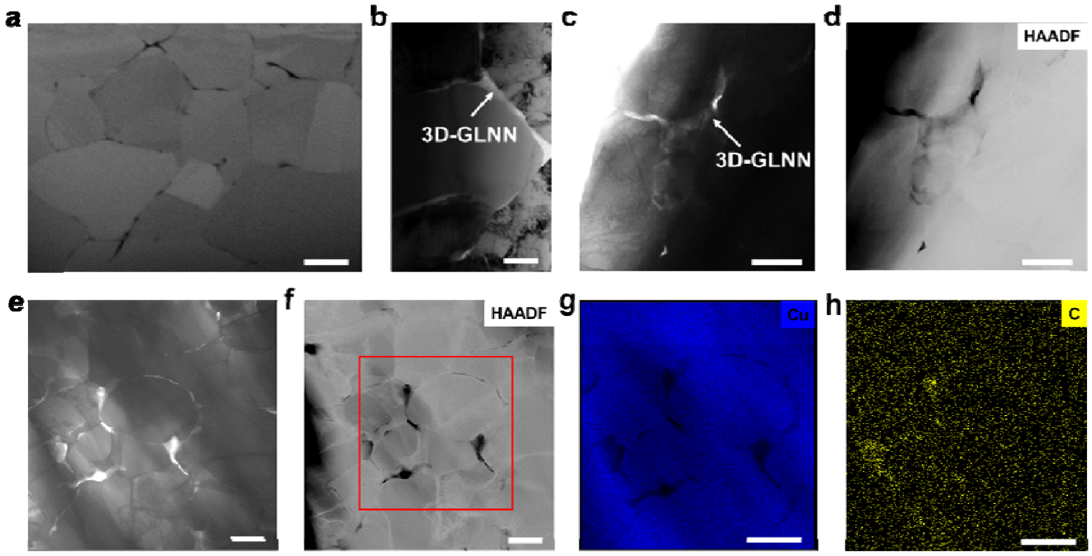

Supplementary Figure 13. Typical microstructure of 3D-GLNN/Cu. a SEM image of the ND-RD plane. Scale bar, 500 nm. **b** Bright-field (BF) TEM image. **c,d,** BF image of Scanning transmission electron microscopy (STEM) mode (**c**) and the corresponding high-angle annular dark-field (HAADF) image (**d**). Scale bar, 200 nm (**b-d**). **e-h** Element mapping of 3D-GLNN/Cu. STEM-BF image (**e**) and the corresponding HAADF image (**f**), and high-resolution energy dispersive Spectroscopy of Cu (**g**) and C (**h**). Scale bar, 1 μm (**e-h**).

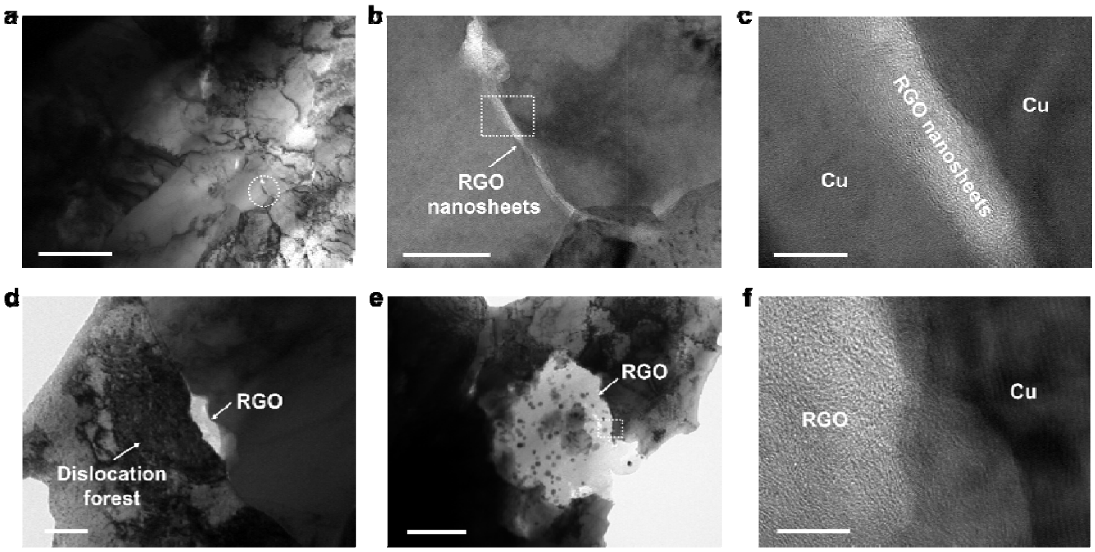

Supplementary Figure 14. TEM images of the typical microstructure of hot-rolled RGO/Cu composites. a-c Low-magnification TEM image showing the micro-level average grain size (**a**) and the corresponding magnified image taken near the grain boundaries (**b**), and HRTEM image of the Cu-RGO-Cu interface corresponding to the marked area (**c**). Scale bar, 1 μm (**a**); 100 nm (**b**); 10 nm (**c**). **d** Dislocation forest near the RGO/Cu interface. Scale bar, 200 nm. **e,f,** Isolated island-like distribution feature of RGO in the composites (**e**) and the corresponding HRTEM images taken near the interface (**f**). Scale bar, 1 μm (**e**); 10 nm (**f**).

Supplementary Table 2. Comparison of mechanical properties data of nanocarbon reinforced metal matrix composites in available literatures.

Materials	f (vol. %)	$\sigma_{UTS}(C)$ (MPa)	$\sigma_{YS}(M)$ (MPa)	R (%)	FE (%)	FE Retention (%)	Reference
3D-GLNN/Cu	0.387	319	202	149.7	25	73	This work
RGO/Cu-lamellar	1.2	308	218	34.4	26	115	1
RGO/Cu-lamellar	1.8	362	258	22.4	7.1	55	2
RGO/Cu-lamellar	0.8	705	494	53.4	11	96	3
CNT-RGO/Al-lamellar	1.5	415	250	44	5.9	34	4
RGO/Al-lamellar	1.5	302	201	50.2	5.3	98	5
CNTs/Al-lamellar	2	435	290	25	6	35	6
CNTs/Cu-lamellar	1.04	283	175	59.3	3	24	7
CNTs/Cu-lamellar	2.5	460	275	26.9	20.9	54	8
CNTs/Al-homogenous	1.3	368	168	91.6	16	69	9
CNTs/Al-homogenous	1.5	182	117	42.7	15.2	61	10
CNTs/Al-homogenous	2	201	134	25	22	93	11
FLG/Al-homogenous	0.7	450	280	86.7	3	11	12
RGO/Cu-homogenous	1	320	250	28	24	63	13
RGO/Cu-homogenous	1	251	190	32.1	18	55	14
GNR/Cu-homogenous	3	295	150	32.2	4.5	90	15
MLG/Cu-homogenous	1	425	384	10.7	16.4	65	16
CNTs/Cu-homogenous	3	398	500	8.5	12.2	59	17
CNTs/Cu-homogenous	3	865	434	33.1	4.2	21	18
CNTs/Cu-homogenous	0.5	560	450	48.9	8	103	19

Supplementary Note 3. Discussion on the tensile deformation of pure Cu, RGO/Cu and 3D-GLNN/Cu

The engineering stress-strain curve of 3D-GLNN/Cu in Supplementary Figure 15 were divided into four distinct regimes (**I-IV**) by short dash lines as a convenience to readers: **(I)** elastic loading up to the yield point (~0.2%); **(II)** strain hardening from the yield point to about 2% strain, in which process the dislocation interactions and multiplication occurred and led to dislocation pile-up inside Cu grains; **(III)** steady flow at a nearly constant stress which indicates the dislocation in regime **II** may be quickly balanced by dislocation annihilation at the 3D-GLNN/Cu interface; **(IV)** elongation after peak stress until final fracture, which is dominated by the interaction between 3D-GLNN and the micro-cracks. By comparison, the stress curve of pure Cu displayed a different feature. It contains only three typical process without regime **III**. Inversely, a much larger regime **II** indicates that the tensile behavior of pure Cu reflects a typical strain-hardening feature of recrystallized Cu with coarse grain sizes (as shown in Supplementary Figure 11b and c). After reaching the peak stress, the gauge area reduced quickly due to the necking-related failure mechanism in regime **IV**. For RGO/Cu, it only has two typical regimes of **I** and **II**. In view of the relatively weak restriction of grain boundary movement in RGO/Cu with randomly-distributed 2D RGO nanosheets, an obvious recrystallization also occurred and resulted in a relatively low dislocation density in the hot-rolled samples. So the curve trend in regime **II** for RGO/Cu was similar to that of pure Cu. It should be noticed that the inevitable agglomeration of RGO nanosheets and also the weak RGO/Cu interface

induced the formation of massive micro-cracks in the bulk composites after the initiation of plastic deformation. A pre-fracture occurred as a result of quick crack propagation.

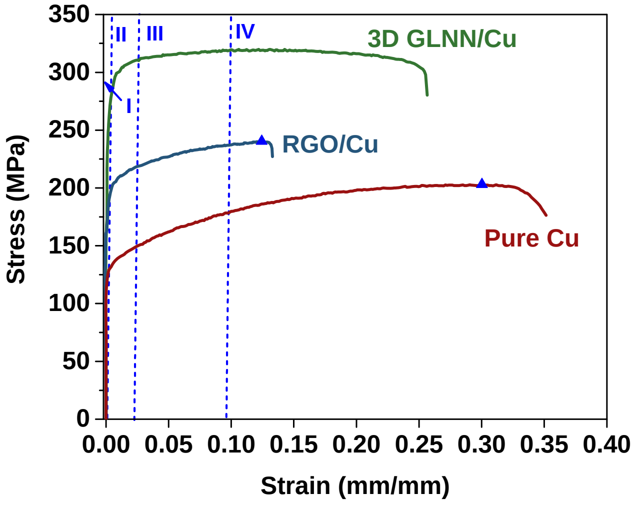

Supplementary Figure 15. Tensile stress-strain curves of pure Cu, RGO/Cu and 3D-GLNN/Cu. The upside-down triangles in blue indicates the peak stress point in the curves of RGO/Cu and pure Cu, respectively.

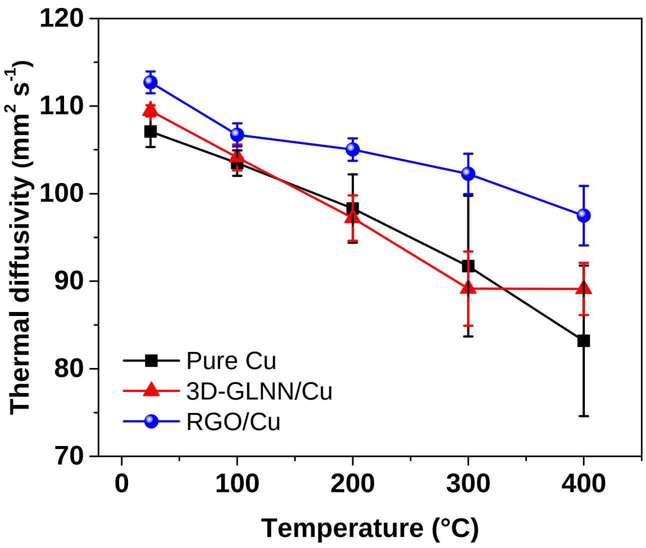

Supplementary Figure 16. Temperature versus thermal diffusivity plot of pure Cu, RGO/Cu and 3D-GLNN/Cu.

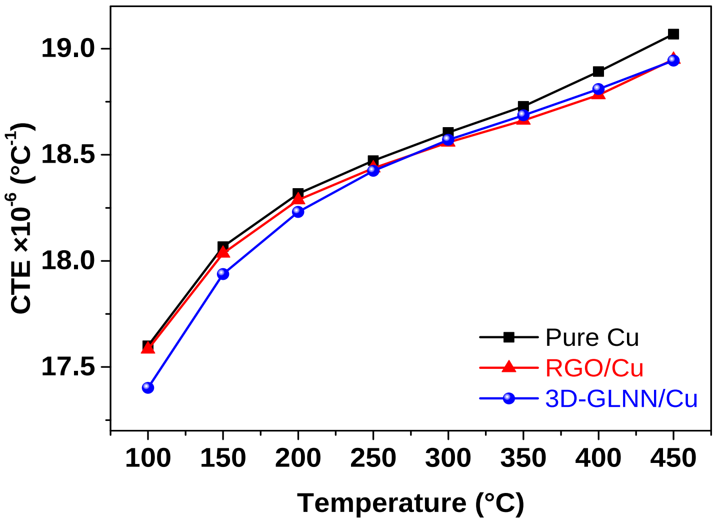

Supplementary Figure 17. Temperature versus CTE of pure Cu, RGO/Cu and 3D-GLNN/Cu composites.

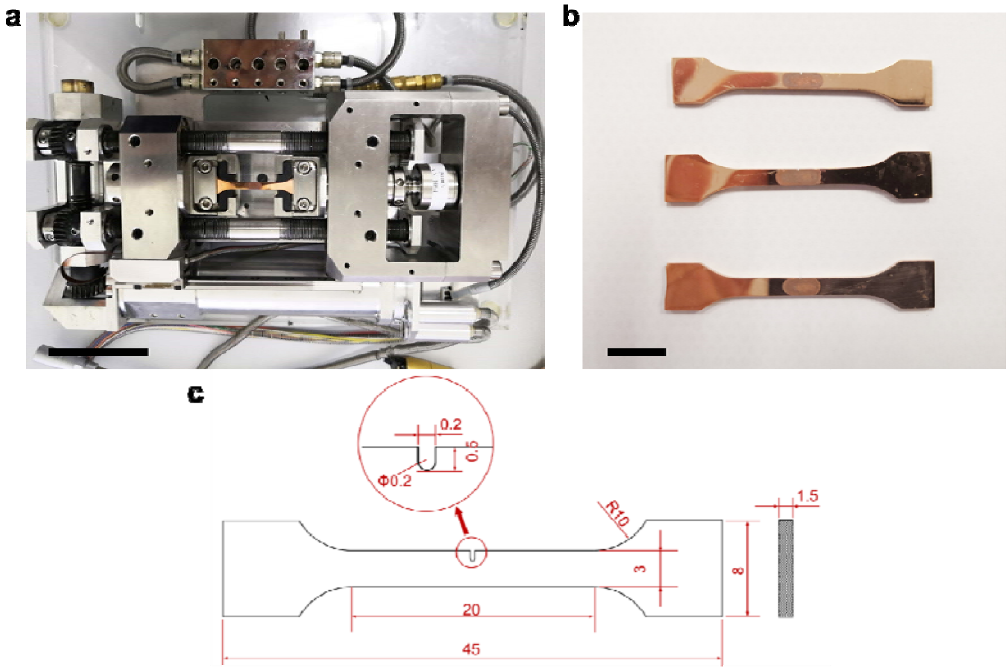

Supplementary Figure 18. Device and specimens for in-situ tensile test. a Photograph of deformation device system installed in the SEM chamber. Scale bar, 50 mm. **b** Photograph of dog-bone specimen with notch on the one side for in-situ tensile deformation. Scale bar, 10 mm. **c** The specimen size for in-situ tensile deformation.

Supplementary Table 3. Experimental data for thermal conductivity measurements of Cu, 3D-GLNN/Cu and RGO/Cu composites.

Sample	Temp. (°C)	ρ (g cm^{-3})	C_p (J g^{-1}K^{-1})	$\alpha_{//}$ (mm^2 s^{-1})	$\alpha_{\perp}$ (mm^2 s^{-1})	$K_{//}$ (W m^{-1} K^{-1})	$K_{\perp}$ (W m^{-1} K^{-1})
Cu	25	8.903	0.395	107.082	111.322	378	394
	100		0.399	103.482	--	368	--
	200		0.398	98.303	--	348	--
	300		0.397	91.721	95.391	325	350
	400		0.398	83.182	--	295	--
3D-GLNN/Cu	25	8.767	0.418	112.706	102.236	413	375
	100		0.430	106.720	--	402	--
	200		0.442	105.026	--	407	--
	300		0.454	102.252	87.644	407	349
	400		0.466	97.489	--	398	--
RGO/Cu	25	8.820	0.391	109.447	105.046	377	362
	100		0.397	104.134	--	365	--
	200		0.404	97.209	--	346	--
	300		0.41	89.149	89.841	322	325
	400		0.416	89.114	--	327	--

Supplementary Table 4. Comparison of thermal conductivity data of nanocarbon reinforced copper matrix composites reported in the literatures.

Materials	f (vol. %)	$K_{\parallel}$ (W m^{-1} K^{-1})	$K_{\perp}$ (W m^{-1} K^{-1})	K_M (W m^{-1} K^{-1})	$K_{\perp} / K_{\parallel}$	η %	Ref.
3D-GLNN/Cu	0.387	413	375	378	0.91	23.9	This work
RGO/Cu-lamellar	5	355	280	350	0.79	2.9	[20]
	12	368	190		0.52	0.4	
	20	418	167		0.40	1.0	
GNP-Cu/aligned	2	505	~380	381	0.75	16.3	[21]
N-doped GNS-Cu film	30	542.9	--	333.5	--	2.1	[22]
RGO-Cu/homogenous	1.2	395	395	360	--	8.1	[23]
RGO-Cu/homogenous	1	360	360	350	--	2.9	[24]
CNTs-Cu/homogenous	1	360	360	348.7	--	3.2	[25]
Aligned CNTs-Cu film	0.26	406.8	--	400	--	6.5	[7]
Graphite flake-Cu/aligned	40	449	71	375	0.16	0.49	[26]
Graphite flake-Cu/aligned	59	457	79	377	0.17	0.4	[27]

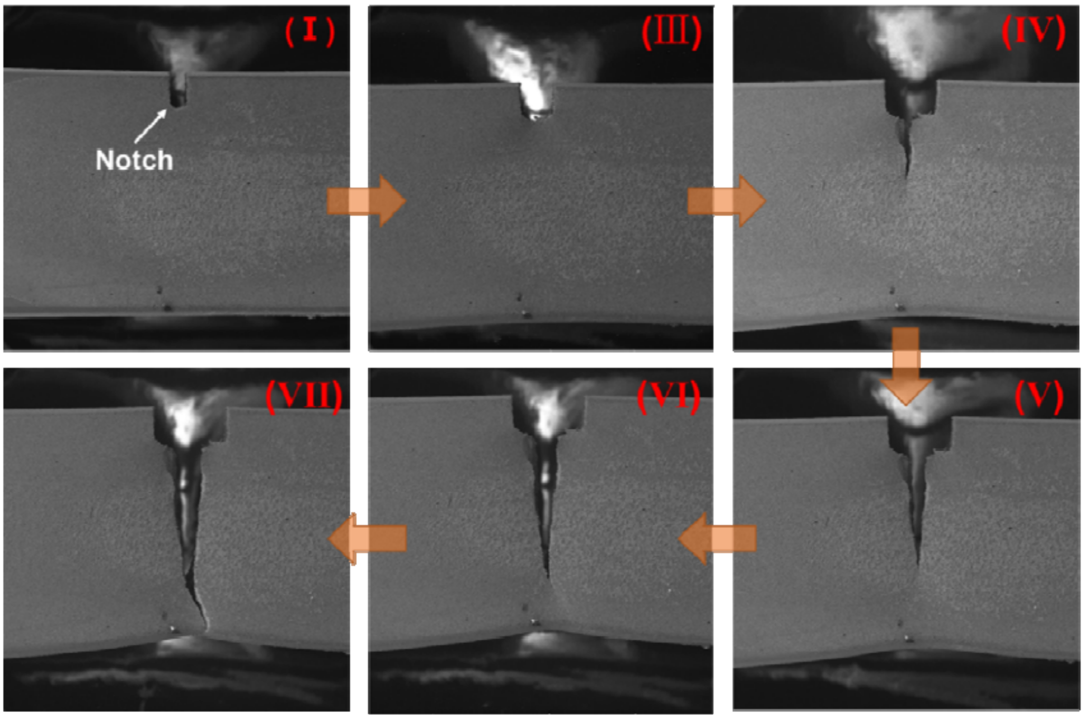

Supplementary Figure 19. SEM images of the crack revolution at the interrupted (I)-(VI) stages of 3D-GLNN/Cu marked in Figure 6a.

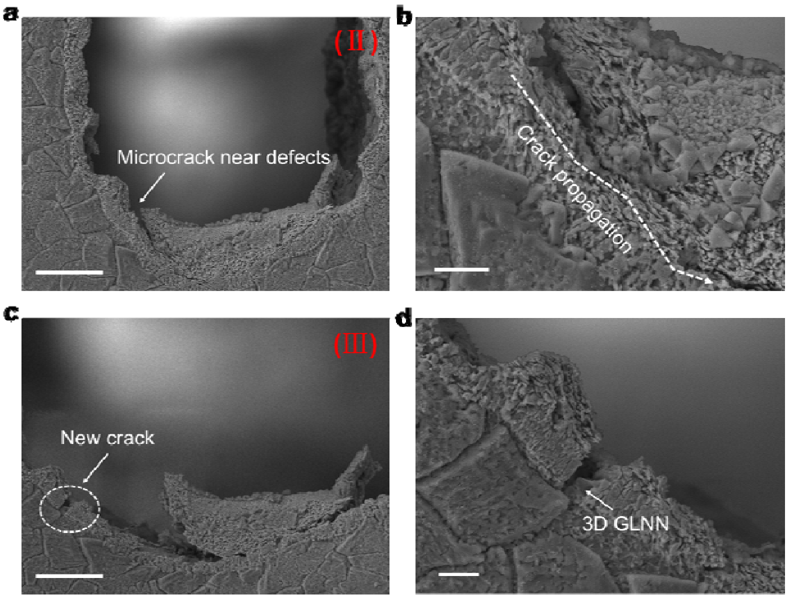

Supplementary Figure 20. Snapshots of the in-situ tensile tests of 3D-GLNN/Cu in the initiation period. a,b, SEM images of the crack tip of (II) stage in Figure 6a (**a**) and the corresponding enlarged views (**b**) of 3D-GLNN/Cu. Scale bar, 50 μm (**a**); 10 μm (**b**). **c,d,** SEM images of the crack tip of (III) stage in Figure 6a (**c**) and the corresponding enlarged views (**d**) of 3D-GLNN/Cu. Scale bar, 50 μm (**c**); 10 μm (**d**).

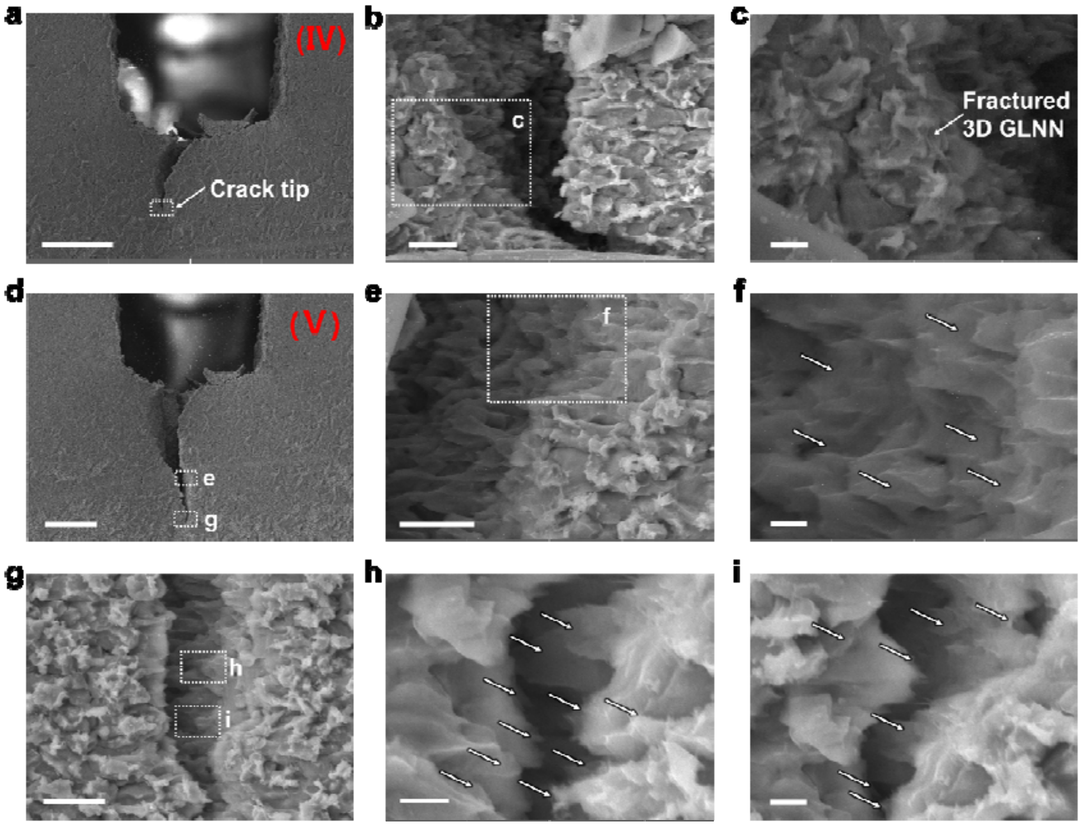

Supplementary Figure 21. Snapshots of the in-situ tensile tests of 3D-GLNN/Cu in the propagation stage. a-c SEM image of the crack tip of (IV) stage in Figure 6a (**a**) and the corresponding enlarged view of the crack tip (**b**), and morphology of fractured 3D-GLNN of the marked area (**c**). Scale bar, 200 μm (**a**); 5 μm (**b**); 2 μm (**c**). **d-i** SEM image of the crack tip of (V) stage in Figure 6a (**d**), the enlarged view of the area behind the crack tip (**e**) and the corresponding marked area showing the fractured 3D-GLNNs (**f**), the enlarged view in the front of crack tip (**g**) and the corresponding marked areas in the upper position (**h**) and in the lower position (**i**) in which the arrows indicated the fractured 3D-GLNNs. Scale bar, 200 μm (**d**); 5 μm (**e**, **g**); 1 μm (**f**, **h** and **i**).

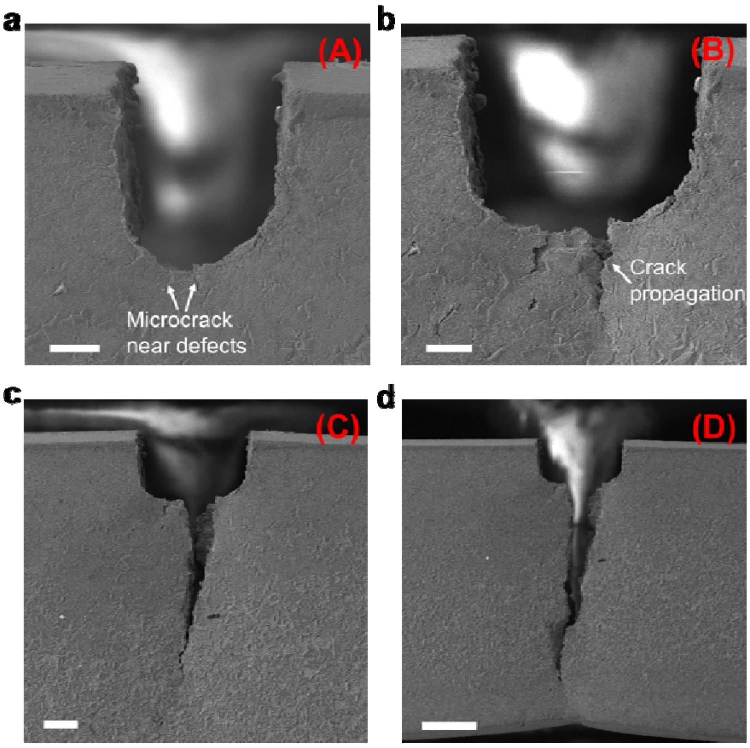

Supplementary Figure 22. Crack evolution of RGO/Cu. a-d SEM images of the crack revolution at the interrupted (**a)** (A), (**b**) (B), (**c**) (C) and (**d**) (D) stages of RGO/Cu marked in Figure 6a. Scale bar, 100 μm (**a-c**); 500 μm (**d**).

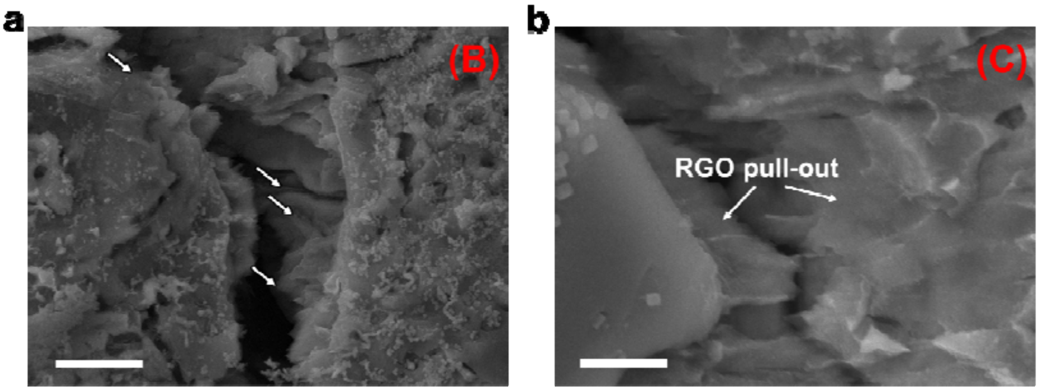

Supplementary Figure 23. Snapshots of in-situ tensile tests of RGO/Cu. a SEM image of the crack tip in the propagation stages of (B). The arrows marked the positions of RGO nanosheets. Scale bar, 5 μm. **b** SEM image of the crack tip in the propagation stages of (C). Scale bar, 2 μm.

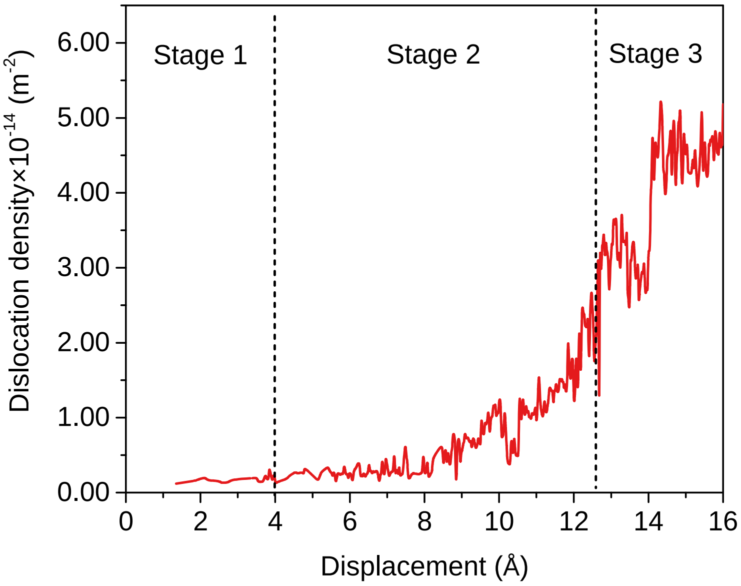

Supplementary Figure 24. Dislocation density versus displacement curve of 3D-GLNN/Cu during the pull-out process. The dislocation density was calculated by:

$$\rho_D = \left(\frac{L_{total}}{V_m} \right) \tag{1}$$

Where L_{total} is the total dislocation length, which was derived from the data analysis in Ovito; V_m is the volume of the simulated copper matrix.

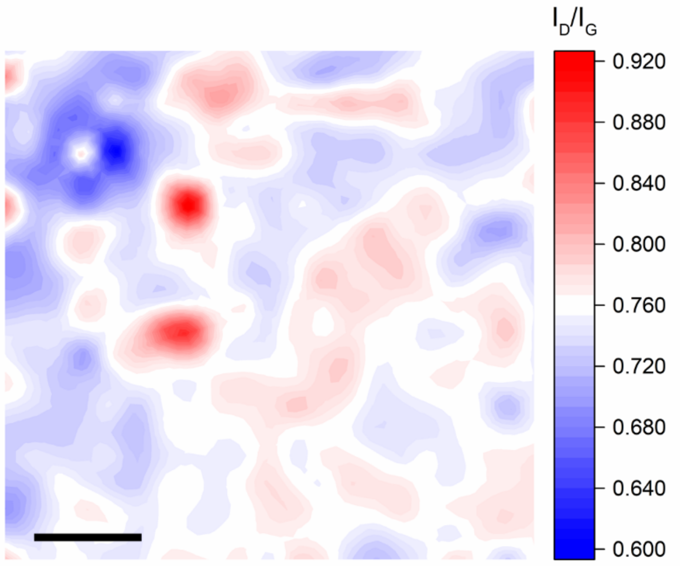

Supplementary Figure 25. Contour plots of the intensity ratio of the D band to the G band for 3D-GLNN in a 10 μm × 10 μm area. Scale bar, 2 μm.

Supplementary Note 4. The detailed derivation for the calculation of the interfacial shear stress of the composites.

The strengthening effect of graphene/Cu composites in this work could be attributed to three aspects, namely grain refinement strengthening or Hall-Petch strengthening (Δ_{H-P}), geometrically necessary dislocations (GNDs) strengthening (Δ_{GNDs}) and load transfer strengthening (Δ_{LT}) mechanisms, which could be expressed as below:

$$\sigma_c = \sigma_m + \Delta_{H-P} + \Delta_{GNDs} + \Delta_{LT} \quad (2)$$

Hall-Petch strengthening is a remarkable strengthening mechanism in nano/ultra-grained MMCs which is related to the increased lattice dislocation blocking with increased numbers of grain boundaries.[28] Δ_{H-P} could be calculated in accordance with the Hall-Petch relationship:

$$\sigma_m = \sigma_0 + kd^{-1/2} \quad (3)$$

where σ_0 and k are empirical constants; k is about 0.07 MPa·m^{-1} for Cu.[18] σ_0 is the frictional stress, k is a measure of the resistance to dislocation caused by grain boundaries. σ_m is the yield strength of the matrix and d is the grain size of the matrix. Δ_{H-P} could be obtained by evolving Equation 3 into the form below:

$$\Delta_{H-P} = \sigma_{m'} - \sigma_m = k\left(d_c^{-1/2} - d_m^{-1/2}\right) \quad (4)$$

where $\sigma_{m'}$ is the improved yield stress caused by Hall-Petch strengthening, d_c is the average grain size of the hot-rolled composites (1.7 μm for 3D-GLNN/Cu and 9.7 μm for RGO/Cu) and d_m is the average grain size of the hot-rolled pure Cu (70.0 μm). The grain size data was obtained from the EBSD statics results in

Supplementary Figure 7b, e and Supplementary Figure 8a, respectively. Therefore, Δ_{H-P} was estimated as 45.3 MPa for 3D-GLNN/Cu and 31.2 MPa for RGO/Cu.

The geometrically induced necessary dislocation strengthening was induced by the dislocation stacking near the interface during the fabrication or deformation progress due to the mismatch between the elastic modulus and CTEs between the matrix and reinforcement. The contribution of GNDs strengthening could be described as below [29]:

$$\Delta_{GND} = \alpha G b \sqrt{\frac{8V_f \varepsilon_y}{b d_e} + \frac{12 V_f \Delta CTE \Delta T}{b(1-V_f) d_e}} \tag{5}$$

Where α is a constant of 1.25; G is the shear modulus of Cu matrix, ~44.8 GPa[15]; ε_y is the yield elongation, the value of which is 0.2%; b is the burgers vector of copper, ~0.256 nm; d_e is the equivalent diameter of the geometric size of graphene. For 3D-GLNN, the value of d_e was reckoned as the average wall size of 2 μm; For RGOs, the value of d_e was considered as the average size of RGO basal plane of about 3.5 μm; ΔCTE is the CTE mismatch between graphene and copper, which is calculated as 23×10^{-6} K^{-1} (CTE value for graphene: ~10^{-6} K^{-1} and copper 24×10^{-6} K^{-1}); ΔT is the temperature difference between the highest temperature during hot deformation (1073 K) and the room temperature (298 K), ~775 K; Δ_{GND} was then estimated as 19.0 MPa for 3D-GLNN/Cu and 14.3 MPa for RGO/Cu.

For load transfer strengthening, the 3D-GLNN/Cu and RGO/Cu were both considered to be simplified into discontinuous and aligned graphene plates reinforced composites in order to compare the value of interfacial shear stress more easily. In this way, the interfacial shear stress could be estimated based on the ideal shear-lag theory.

Shin et al. proposed a modified shear-lag model considering the plate-like geometry of graphene, which was written as[12]:

$$\sigma_c = V_f (\frac{S}{A}) \left(\frac{\tau}{2} \right) + \sigma_m V_m \tag{6}$$

Where S and A are the size of the interface in basal plane and cross section of graphene, respectively; For 3D-GLNN, the S value could be estimated as 2×2×2 μm^2; A was estimated as 2×2×0.0003 μm^2; the S value for RGO was 2×3.5×3.5 μm^2 and A value was estimated as 2×3.5×0.0005 μm^2; τ is the interfacial shear stress; σ_m is the yield strength and V_m is the volume fraction of the matrix. Δ_{LT} could be obtained by subtracting the contribution of Δ_{GND} and Δ_{H-P} from the total increase in yield strength (Δ_{YS}) and the interfacial shear stress could be thereby calculated as:

$$\Delta_{LT} = \Delta_{YS} - \Delta_{H-P} - \Delta_{GNDs} \tag{7}$$

$$\Delta_{LT} = \sigma_c - \sigma_m = V_f (\frac{S}{A}) \left(\frac{\tau}{2} \right) - \sigma_m V_f = V_f [(\frac{S}{A}) \left(\frac{\tau}{2} \right) - \sigma_m] \tag{8}$$

$$\tau = \left(\frac{\Delta_{LT}}{V_f} + \sigma_m \right) \frac{2A}{S} \tag{9}$$

For 3D-GLNN/Cu, Δ_{LT} was estimated as 110.7 MPa and the corresponding interfacial shear stress was obtained as 86.2 MPa; While Δ_{LT} for RGO/Cu was 35.5 MPa and the interfacial shear stress was determined as 26.5 MPa.

Supplementary References

1. Xiong, D. et al. Graphene-and-copper artificial nacre fabricated by a preform impregnation process: Bioinspired Strategy for Strengthening-Toughening of Metal Matrix Composite. *ACS Nano* **9**, 6934-6943 (2015).

2. Li, Z. et al. Graphene quality dominated interface deformation behavior of graphene-metal composite: The defective is better. *Inter.J. Plasticity* (2018).

3. Li, Z. et al. Regain strain-hardening in high-strength metals by nanofiller incorporation at grain boundaries. *Nano Lett.* **18**, 6255-6264 (2018).

4. Li, Z. et al. Synergistic strengthening effect of graphene-carbon nanotube hybrid structure in aluminum matrix composites. *Carbon* **95**, 419-427 (2015).

5. Li, Z. et al. Enhanced mechanical properties of graphene (reduced graphene oxide)/aluminum composites with a bioinspired nanolaminated structure. *Nano Lett.* **15**, 8077-8083 (2015).

6. Jiang, L., Li, Z., Fan, G., Cao, L. & Zhang, D. The use of flake powder metallurgy to produce carbon nanotube (CNT)/aluminum composites with a homogenous CNT distribution. *Carbon* **50**, 1993-1998 (2012).

7. Shuai, J., Xiong, L., Zhu, L. & Li, W. Enhanced strength and excellent transport properties of a superaligned carbon nanotubes reinforced copper matrix laminar composite. *Compos Part A: Appl. S.* **88**, 148-155 (2016).

8. Liu, J. et al. Mechanical properties and failure mechanisms at high temperature in carbon nanotube reinforced copper matrix nanolaminated composite. *Compos Part A: Appl. S* **116**, 54-61 (2019).

9. Chen, B. et al. Length Effect of Carbon Nanotubes on the Strengthening Mechanisms in Metal Matrix Composites. *Acta Mater.* **140**, 317-325 (2017).

10. Zhou, W., Yamaguchi, T., Kikuchi, K., Nomura, N. & Kawasaki, A. Effectively enhanced load transfer by interfacial reactions in multi-walled carbon nanotube reinforced Al matrix composites. *Acta Mater.* **125**, 369-376 (2017).

11. So, K.P. et al. Ton-scale metal – carbon nanotube composite: The mechanism of strengthening while retaining tensile ductility. *Extreme Mech. Lett.* **8**, 245-250 (2016).

12. Shin, S.E., Choi, H.J., Shin, J.H. & Bae, D.H. Strengthening behavior of few-layered graphene/aluminum composites. *Carbon* **82**, 143-151 (2015).

13. Hwang, J. et al. Enhanced mechanical properties of graphene/copper nanocomposites using a molecular-level mixing process. *Adv. Mater.* **25**, 6724-6729 (2013).

14. Chu, K., Wang, J., Liu, Y. & Geng, Z. Graphene defect engineering for optimizing the interface and mechanical properties of graphene/copper composites. *Carbon* **140**, 112-123 (2018).

15. Yang, M., Weng, L., Zhu, H., Fan, T. & Zhang, D. Simultaneously enhancing the strength, ductility and conductivity of copper matrix composites with graphene nanoribbons. *Carbon* **118**, 250-260 (2017).

16. Kim, W.J., Lee, T.J. & Han, S.H. Multi-layer graphene/copper composites: Preparation using high-ratio differential speed rolling, microstructure and mechanical properties. *Carbon* **69**, 55-65 (2014).

17. Yoo, S.J., Han, S.H. & Kim, W.J. A combination of ball milling and high-ratio

differential speed rolling for synthesizing carbon nanotube/copper composites. *Carbon* **61**, 487-500 (2013).

18. Wang, H. et al. Synergistic strengthening effect of nanocrystalline copper reinforced with carbon nanotubes. *Sci. Rep.***6**, 26258 (2016).

19. Arnaud, C. et al. High strength - High conductivity double-walled carbon nanotube -Copper composite wires. *Carbon* **96**, 212-215 (2016).

20. Chu, K. et al. Largely enhanced thermal conductivity of graphene/copper composites with highly aligned graphene network. *Carbon* **127**, 102-112 (2018).

21. Nazeer, F. et al. Thermal and mechanical properties of copper-graphite and copper-reduced graphene oxide composites. *Compos. Part B: Eng.***163**, 77-85 (2019).

22. Zheng, L. et al. N-doped graphene-based copper nanocomposite with ultralow electrical resistivity and high thermal conductivity. *Sci. Rep.* **8,** 9248 (2018).

23. Gao, X. et al. Mechanical properties and thermal conductivity of graphene reinforced copper matrix composites. *Powder Technol.* **301**, 601-607 (2016).

24. Chu, K. et al. Interface and mechanical/thermal properties of graphene/copper composite with Mo 2 C nanoparticles grown on graphene. *Compos Part A: Appl. S.* **109**, 267-279 (2018).

25. Cho, S., Kikuchi, K. & Kawasaki, A. On the role of amorphous intergranular and interfacial layers in the thermal conductivity of a multi-walled carbon nanotube–copper matrix composite. *Acta Mater.* **60**, 726-736 (2012).

26. Bai, H. et al. Thermal conductivity and mechanical properties of flake graphite/copper composite with a boron carbide-boron nano-layer on graphite surface.

Compos Part A: Appl. S. **106**, 42-51 (2018).

27. Sohn, Y., Han, T. & Han, J.H. Effects of shape and alignment of reinforcing graphite phases on the thermal conductivity and the coefficient of thermal expansion of graphite/copper composites. *Carbon* **149**, 152-164 (2019).

28. Zhu, L. & Lu, J. Modelling the plastic deformation of nanostructured metals with bimodal grain size distribution. *Inter. J. Plasticity* **30-31**, 166-184 (2012).

29. Jiang, L. et al. Toughening of aluminum matrix nanocomposites via spatial arrays of boron carbide spherical nanoparticles. *Acta Mater.* **103**, 128-140 (2016).
